# *Nectopsyche* of Ecuador: a new species from the high Andean páramo and redescription of *Nectopsyche spiloma* (Ross) (Trichoptera: Leptoceridae)

**DOI:** 10.7717/peerj.4981

**Published:** 2018-06-27

**Authors:** Ralph W. Holzenthal, Blanca Rios-Touma

**Affiliations:** 1 Department of Entomology, University of Minnesota—Twin Cities Campus, St. Paul, MN, United States of America; 2 Facultad de Ingenierías y Ciencias Aplicadas. Grupo de Investigación en Biodiversidad, Medio Ambiente y Salud -BIOMAS-, Universidad de las Americas, Quito, Ecuador; 3 Instituto Nacional de Biodiversidad, Quito, Pichincha, Ecuador

**Keywords:** New species, New record, Larva, Taxonomy, Redescription, High elevation, Caddisfly, Neotropics, Andes

## Abstract

The male and female of a new species of long-horned caddisfly, *Nectopsyche paramo*, are described from the high Andes of Ecuador. The new species was found above 4,000 m, representing the highest recorded elevation for a species in the genus. The larval stage of the species is also described. Only a total of 13 larvae were collected during a 17-month sampling program and 11 adults, suggesting that the species is rare. Larvae were found mainly in leaf packs. A male and female were observed in a mating swarm ca. 3 m above a stream during late afternoon. In addition, we redescribe the adult male of *Nectopsyche spiloma* (Ross), previously known from Ecuador from unsubstantiated literature records.

## Introduction

The long-horned caddifly genus *Nectopsyche* is restricted to the New World, with species occurring from southern and western Canada, throughout the United States, Mexico, the Caribbean, Central America, and South America, including Chile. While some species are somber colored like most caddisflies, many members of the genus are brightly patterned due to the presence of colored hairs and scales on the wings, often with iridescent or metallic tones (Holzenthal, 1995). Forty-eight of the 58 described species of *Nectopsyche* occur in the Neotropics, but there are many new species in existing collections waiting to be described, and probably many more to be discovered in nature (Holzenthal & Calor, 2017). Types of seven of the described Neotropical species are lost or unidentifiable, rendering their identity equivocal. The senior author is engaged in a comprehensive revision of the Neotropical *Nectopsyche*.

Of the 18 species reported from the Andean countries, 11 have been reported from Ecuador, with an altitudinal distribution from 240 to 1,600 m above sea level (asl) (Holzenthal & Calor, 2017; Ríos-Touma et al., 2017). *Nectopsyche* larvae in Andean streams have been reported to be shredders, mainly eating coarse particulate organic matter (Ríos-Touma, Encalada & Prat Fornells, 2009). They show a preference for slowly flowing areas and are highly susceptible to predation by the exotic trout *Oncorhynchus mykiss* (Ríos-Touma, Encalada & Prat Fornells, 2011; Vimos et al., 2015). In this paper, we describe the adults and larva of a new species from the high Andean páramo of Ecuador, and present some information on the biology of the new species. Also we redescribe the male genitalia and provide a color illustration of the adult male of *Nectopsyche spiloma* (Ross, 1944), a species distributed from central North America to Perú, and provide substantiated records of it from Ecuador for the first time.

## Material and Methods

### Specimen preparation

Adult specimens were collected at UV fluorescent lights placed adjacent to streams. Lights were hung in front of a white bed sheet and powered from a small 12 volt, sealed, lead acid battery. Temperatures at the start of collecting were near 2–4 °C. In total, only three adults were collected at light. These were captured in cyanide kill jars and pinned the following morning. Other adults were netted during the early evening and were either pinned (two specimens) or placed in 80% ethyl alcohol (six specimens).

Additionally, we collected 13 larvae from Quebrada Saltana between 2009 to 2012. Three larvae were collected using a modified Hess sampler of 20.3 cm diameter. Ten larvae were recovered from coarse mesh bags filled with local *Alnus acuminata* leaves used for a decomposition experiment (Pontón Cevallos, 2012). These larvae were measured using a Zeiss Stereo Discovery V12 microscope with an AxioCam ICc5 camera and using the software AxionVision SE64 Re 1.4.9.1 (Oberkochen, Germany).

Adult specimens were prepared and examined following standard methods for pinned and alcohol preserved material (Blahnik & Holzenthal, 2004; Blahnik, Holzenthal & Prather, 2007). Length of forewing was measured from base to apex, and is presented as the range followed by the mean and number of specimens measured. Width of eye and interocular distance were measured below the antennal scape and above insertion of the maxillary palps at the middle of eye and is presented as a simple ratio (see Ross, 1944, figs. 759–761 for orientation). Male genitalia were soaked in 85% lactic acid heated to 125 °C for 20 min to dissolve internal soft tissues. An Olympus BX41 compound microscope (Tokyo, Japan) outfitted with a drawing tube was used to examine specimens and to aid the rendering of detailed pencil drawings of genitalic structures. Pencil sketches were scanned and placed in Adobe Illustrator (Creative Cloud version) to serve as a template for vector illustrations. Morphological terminology follows that of Holzenthal (1995). Each specimen was affixed with a barcode label (4-mil polyester, 8 × 14 mm, code 49) bearing a unique alphanumeric sequence beginning with the prefix UMSP to serve as a specimen identifier for upload of collection and specimen data to the University of Minnesota Insect Collection (UMSP) database.

Types of the new species and other material examined are deposited in the University of Minnesota Insect Collection, St. Paul, Minnesota, USA (UMSP), the Museo Ecuatoriano de Ciencias Naturales, Insituto Nacional de Biodiversidad, Quito, Ecuador (MECN), and the Museo de Ecología Acuática, Universidad San Francisco de Quito, Ecuador (USFQ). This study was performed under the Environmental Ministry of Ecuador study permits: 36-2010-IC-FLO/FAU-DPA-MA, 005-15-IC-FAU-FLO-DNB/MA, and MAE-DNB-CM-2016-0045.

### Study area and larval association

Quebrada Saltana is a first-order pristine stream in the northeastern Andes of Ecuador at an elevation of 3,850 m asl. The stream forms part of the headwaters of the Río Guayllabamba basin and has served as a study site for several investigations in Andean aquatic insect ecology and taxonomy (Holzenthal & Ríos-Touma, 2012; Prat et al., 2017; Vimos et al., 2015). At the study site, it flows through the Reserva de Paluguillo under the administration of the Fondo Para la Protección del Agua (FONAG). Elevation where adults and larvae were collected was 3,850 m asl. Additional specimens of the new species were also collected at high elevations, including a small stream near the village of Porvenir, Pichincha Province (3,361 m asl) and from the Río Culebrillas in the Reserva de Producción Faunística Chimborazo, Bolívar Province (4,134 m asl), making this, to our knowledge, the highest elevation recorded for any species of *Nectopsyche*. All localities are located in the *páramo* in the western Pacific drainage of the Andes of Ecuador. Several studies have provided information on the hydrology, floristics, and environmental impacts affecting this ecosystem (Buytaert et al., 2006; Flores-López et al., 2016; Sklenář & Jørgensen, 1999; Suárez & Medina, 2001).

Association of larvae and adults of the new species was indirect. No specimens were reared, nor were pharate adults or pupae collected. However, adults of only the new species were collected from Quebrada Saltana, the type locality, where larvae of only a single morphotype were also collected during the 17-mo study period.

The electronic version of this article in Portable Document Format (PDF) will represent a published work according to the International Commission on Zoological Nomenclature (ICZN), and hence the new names contained in the electronic version are effectively published under that Code from the electronic edition alone. This published work and the nomenclatural acts it contains have been registered in ZooBank, the online registration system for the ICZN. The ZooBank LSIDs (Life Science Identifiers) can be resolved and the associated information viewed through any standard web browser by appending the LSID to the prefix http://zoobank.org/. The LSID for this publication is: urn:lsid:zoobank.org:pub:D87B7D98-3111-48A1-B100-4835F7179AE2. The online version of this work is archived and available from the following digital repositories: PeerJ, PubMed Central and CLOCKSS.

## Results

### Species description

**Table:** 

**Figure 1:**
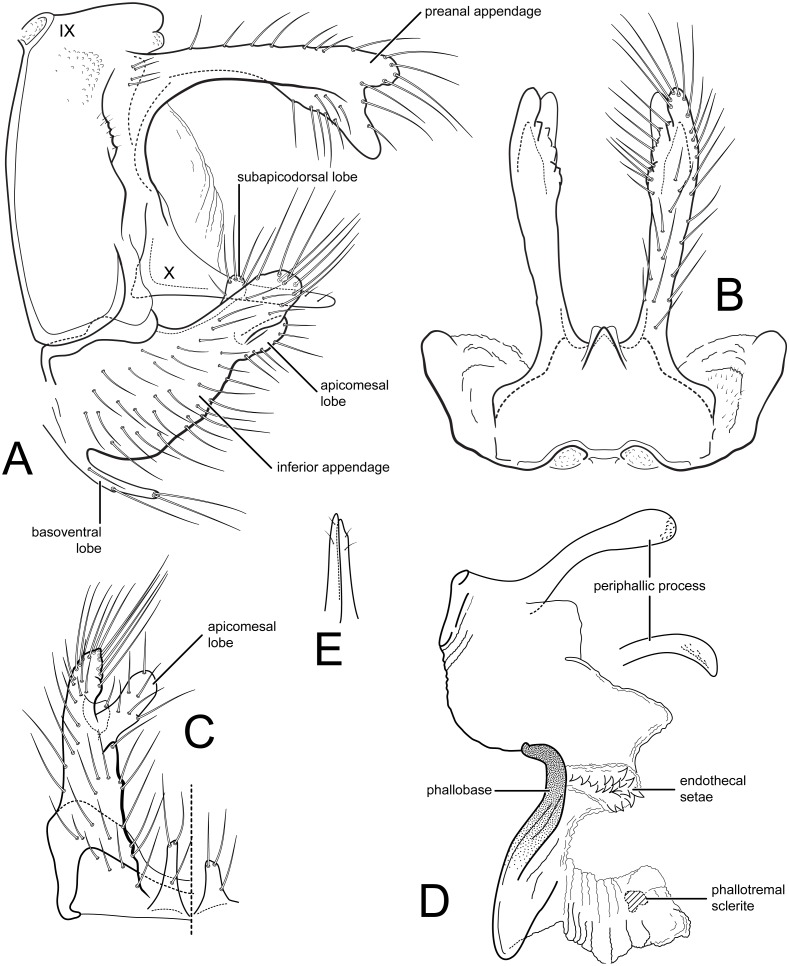
*Nectopsyche paramo*, new species. Male genitalia (A) segments IX-X, lateral (B) segments IX-X, dorsal (C) inferior appendage, ventral (D) phallus, lateral, inset: left periphallic process, dorsal (E) apices segment X lateral processes, dorsal. IX, abdominal segment IX; X, abdominal segment X. Illustrations by RW Holzenthal.

**Figure 2:**
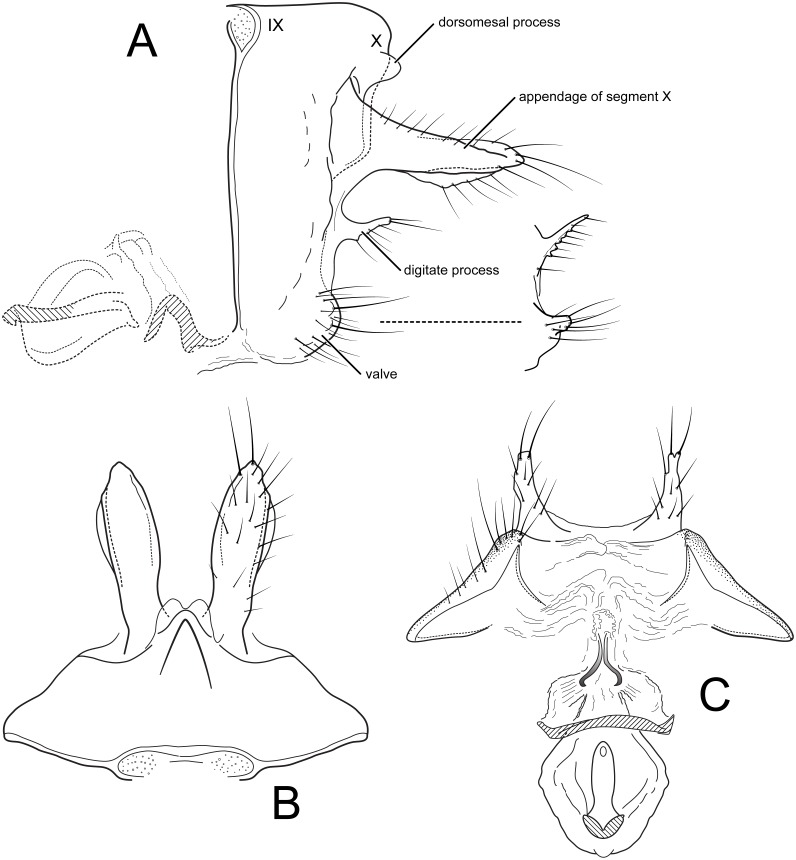
*Nectopsyche paramo*, new species. Female genitalia (A) segments IX-X, vaginal apparatus, lateral, inset: digitate process and appendage of X, lateral, paratype from Porvenir (B) segments IX-X, dorsal (C) segment IX, vaginal apparatus, ventral. IX, abdominal segment IX; X, abdominal segment X. Illustrations by RW Holzenthal.

**Figure 3:**
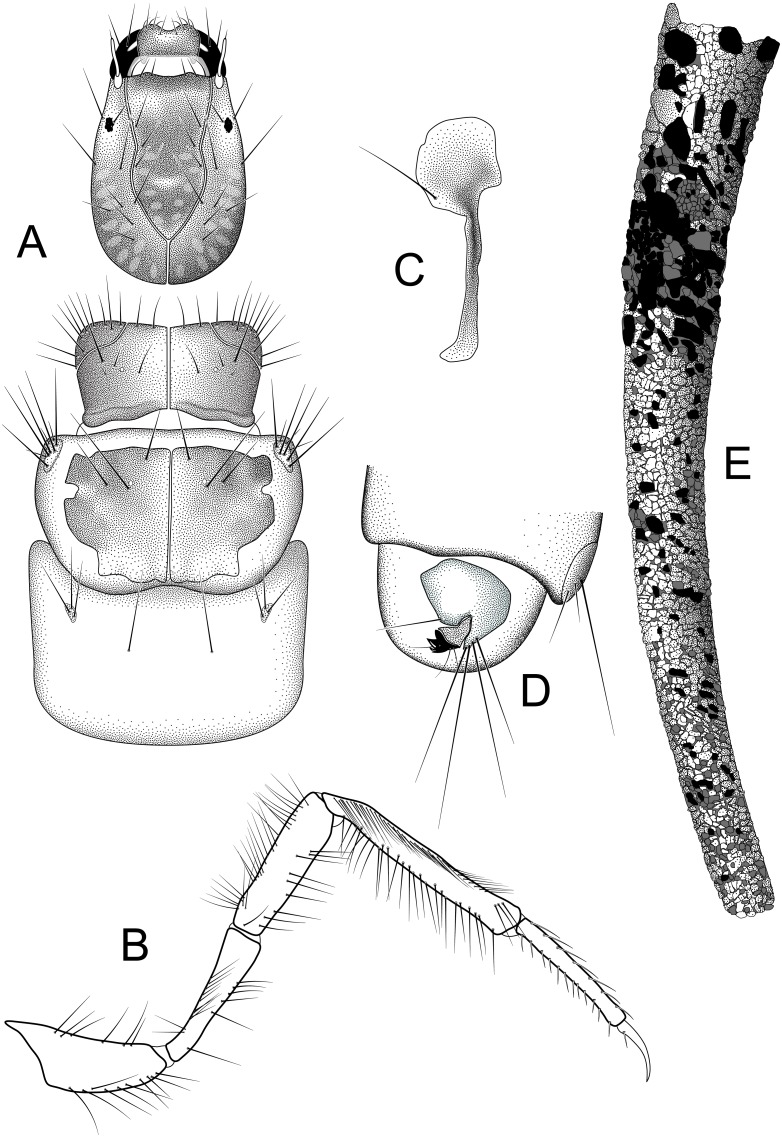
*Nectopsyche paramo*, new species. Larva and case (A) head and thorax, dorsal (B) right hind leg, lateral (C) lateral hump sclerite (D) abdominal segments IX, X, lateral (E) case, lateral. Illustrations by RW Holzenthal.

**Figure 4:**
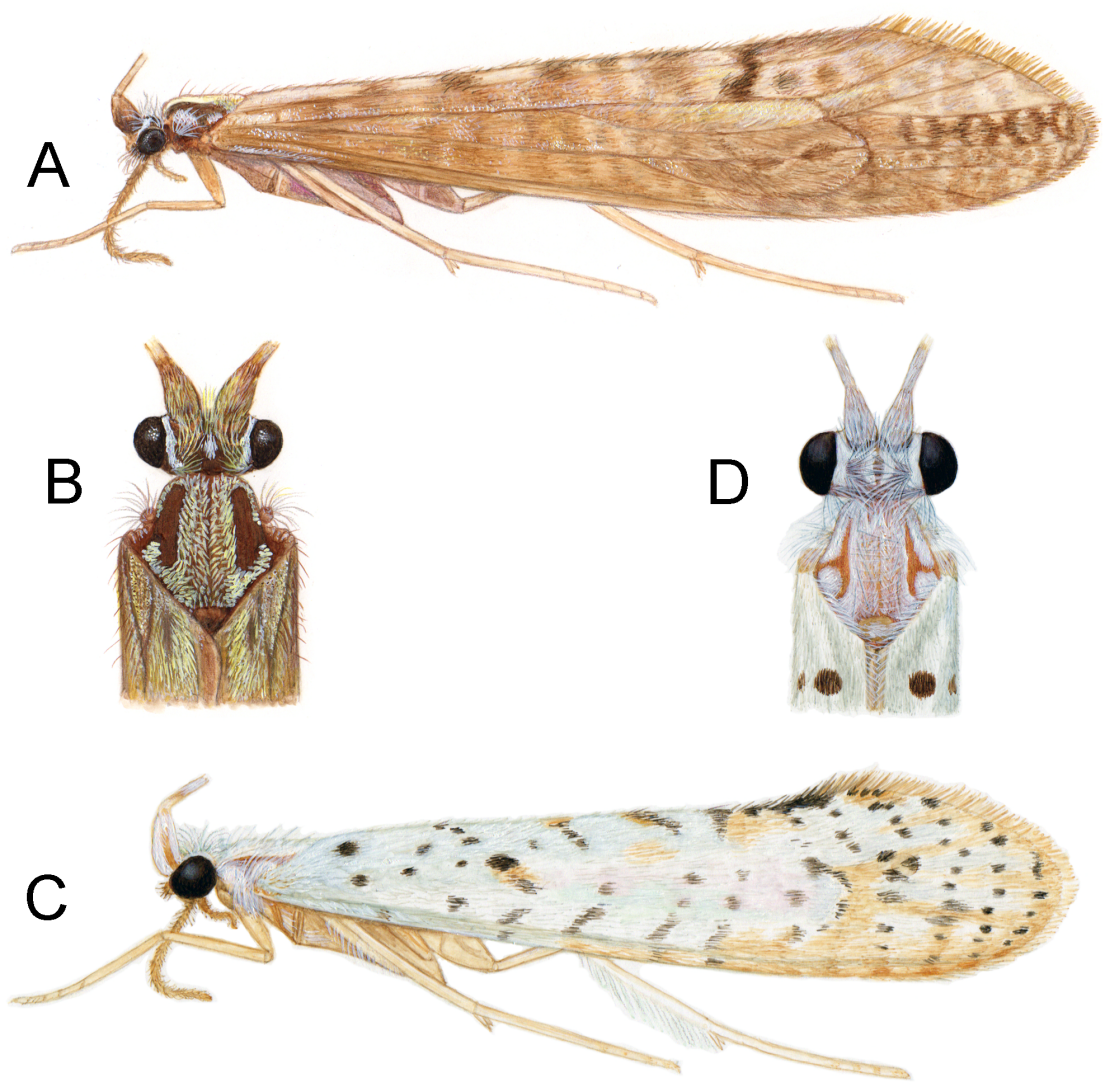
*Nectopsyche* species. *Nectopsyche paramo*, new species (A) lateral (B) head and thorax, dorsal. *Nectopsyche spiloma* (Ross) (C) lateral (D) head and thorax, dorsal. Illustrations by Julie Martinez.

***Nectopsyche paramo*Holzenthal and Ríos-Touma, new species**
Figs. 1, 2, 3, Fig. 4A and 4B

**Diagnosis**. This new species is a member of the *gemma*-group, based on characteristics of the male genitalia, including the forked preanal appendages; the narrow lateral processes of segment X; the distinct periphallic processes without an enlarged apex; and the short spine-like setae present in the endothecal membranes of the phallus. However, the new species completely lacks the distinctive forewing color pattern of yellow hairs, bands of silver metallic scales, and distinctive black spots displayed by other members of the group, including the type species of the genus, *Nectopsyche gemma* Müller (1880[1878]) and several species from Costa Rica, among others (Holzenthal, 1995). Instead, the species has its forewings covered by brown and white hairs distributed in a delicate, somewhat reticulate pattern. It is also much larger than most species in the *gemma*-group. These characters, in combination, render it unique in the genus. (*Note*: forewing hairs are very delicate and easily rubbed off of specimens handled carelessly before pinning; they become completely denuded on alcohol preserved specimens. The forewing hairs are largely intact on the pinned holotype, but variously rubbed on most of the paratypes.)

**Description**. *Male*. Length of forewing 14–15 mm, (14.3, *n* = 6). Head and thorax brown, with yellow-white hairs and scale-like hairs on dorsum; antennae light brown with white hairs on basal flagellomeres, scape covered with yellow-white hairs; labial and maxillary palps brown; legs light brown; forewings light brown with slightly darker brown hairs and white hairs arranged in diffuse round and irregular spots, imparting irregular reticulate pattern; more discrete, circular patches of light brown hairs below (anterior to) vein R4+5, with distinctly lighter hairs above (posterior to) this vein; arculus with darker patch of hairs; anal edge of wing from humerus until about midway to arculus with irregular row of white hairs. Eyes small, width ca. 0.4× interocular distance. *Male genitalia*. Abdominal segment IX rectangular, short, about 2× as high as long; tergum with mid-dorsal protuberance and pair of small oval anterior acrotergites; sternum IX membranous. Preanal appendage long, narrow, setose, forked apically; ventral fork slightly longer than dorsal fork, apex smoothly sclerotized, without apical setae; dorsal fork heavily setose (in male paratype, ventral fork more rounded apically than in holotype). Segment X lateral process thin, very narrow, apex acute, bearing small subapical setae. Inferior appendage broad basally, narrowing apically, with apex rounded in lateral view, and bearing long prominent setae; subapicodorsal setose projection short, rounded (this process slightly longer in male paratype); apicomesal lobe long, narrow, rounded apically, separated from body of inferior appendage, without setae in gap; basoventral process long, widest basally, with long setae. Phallic apparatus with distinct periphallic processes, apices of processes slightly enlarged, pointed, rugose; phallic apodeme enlarged, bulbous, semi-membranous; phallobase trough-like, sinuous, sclerotized; endothecal membranes highly eversible; with paired patches of short spine-like setae present; phallotremal sclerite small, U-shaped in dorsal view.

*Female*. Forewing length 12–13.5 mm (12.9, *n* = 5). Color as in male. *Female genitalia*. Abdominal segment IX rectangular, short, about 3× as high as long; tergum IX with mid-dorsal keel-like protuberance; tergum X with pair of short dorsomesal processes; appendages of segment X long, robust, wide basally, tapering slightly to apex, widest in middle, setose; pair of short, setose, digitate processes present midlaterally, below appendages of X; valves very short, densely setose, rounded, concave on mesal surface and encompassing sternal membranes between them (in the paratype from Porvenir, the digitate processes are narrower and the appendages of segment X are shorter and more acute); vaginal apparatus (spermathecal sclerite complex) with cup-shaped posterior base bearing a sclerotized ridge ventrally and anterior, ovoid part bearing central “keyhole-shaped” structure; vaginal apparatus connected to terminal membranes by narrow, partially sclerotized neck.

*Larva*. Length of body 2.7–10.6 mm (*n* = 13). Structure and setal pattern typical for the genus (Holzenthal, 1995), as described for *N*. *gemmoides* (Flint, 1981). Head: uniformly brown, lighter at middle of frontoclypeal apotome, with faint muscle scars at base of frontoclypeal apotome and in parietal regions; ventral apotome short, triangular; subocular ecdysial line present. Thorax: pronotum uniformly brown, anterior and lateral margins entire, not crenulate or toothed as in some species (Marlier, 1964; Wiggins, 1996); anterolateral corners delimited by ecdysial line; mesonotum with pair of large medial sclerites, light brown basally, with darker pigmentation anteriorly; mesonotal sa3 sclerites small, oval; metanotum with small oval sa3 sclerites; metasternum with pair of long setae; head and thoracic sclerites without diagnostic spots or bars as occur in *N*. *gemmoides* and other species (Glover & Floyd, 2004; Haddock, 1977). Legs long and slender; hind tibia not sub-divided; hind tibia and tarsus with rows of long thin setae as seen in *N*. *gemmoides* and other species (Holzenthal, 1995; Marlier, 1964); setae in dorsal row thinner than those in ventral row. Abdomen: lateral hump sclerite of abdominal segment I with round anterior portion bearing rows of fine microtrichia and with long, narrow, posterolateral, sclerotized extension typical for genus; gills absent; lateral fringe present on segments III-VII; lateral tubercles present on VIII. Tergum IX with lightly sclorotized dorsal plate bearing ca. three pairs of apical setae on posterior edge. Abdominal segment X with lateral sclerite, bearing ca. 4 very long satae on posterior edge; anal claw with two small accessory hooks; ventral band of uniformly minute spines present beside anal opening.

*Larval case*. Length: 3.7–13.3 mm. Slightly curved, tapered, cylindrical; composed of small mineral fragments and larger pieces of plant material in anterior portion, roughly arranged in spiral rows in more mature instars.

**Holotype. Male.** **ECUADOR: Pichincha**: Reserva Paluguillo, Quebrada Saltana, 00.31644°S, 78.22032°W, 3850 m, 15–16.x.2011, Holzenthal, Ríos, Pita (UMSP) (UMSP000098737) [illustrated]. **Paratypes**: Same data as holotype, one male, two females (UMSP); same, except 14-18.iv.2011, B. Ríos-Touma, one male, one female (MECN); same, except 27.iv-3.vi.2011, one female (MECN). **ECUADOR**: **Bolívar**: Río Culebrillas, 1.5082°S 78.8817°W, 4134 m, 22.i.2008, Flowers & Calles, one male (UMSP), one male, one female (MECN). **Pichincha:** small stream near Porvenir, ca. 10 km E Machachi, 00.533408°S, 78.49240°W, el. 3361 m, 2.iii.2017, Ríos-Touma, Holzenthal, Amigo, Huisman, one female (MECN).

### Additional material examined

**ECUADOR: Pichincha**: Reserva Paluguillo, Quebrada Saltana, 00.31644°S, 78.22032°W, 3848 m, 13.i.2010, Ríos-Touma and Gonzaléz, one larva (UMSP); same, various dates, 12 larvae (USFQ).

### Larval habitat

At Quebrada Saltana, *Nectopsyche paramo* was extremely rare. We found only three larvae during a 17-month larval growth and development study at the site, one each in November, 2009, and January and May, 2010. Two of these larvae were found on gravel substrate. In addition, 10 individuals were found in leaf pack samplers during a decomposition experiment performed in the stream during the same period (Pontón Cevallos, 2012).

### Adult flight and mating behavior

On 16 October 2011, at ca. 5:45 pm, a male (the holotype) and a female were observed in a mating flight above Quebrada Saltana. They flew up and down together in an open spiral from about 2-5 m above the stream. Towards the end of the flight, they copulated in the air and fell to the ground still *in copula*, where they were captured by hand.

**Table:** 

**Figure 5:**
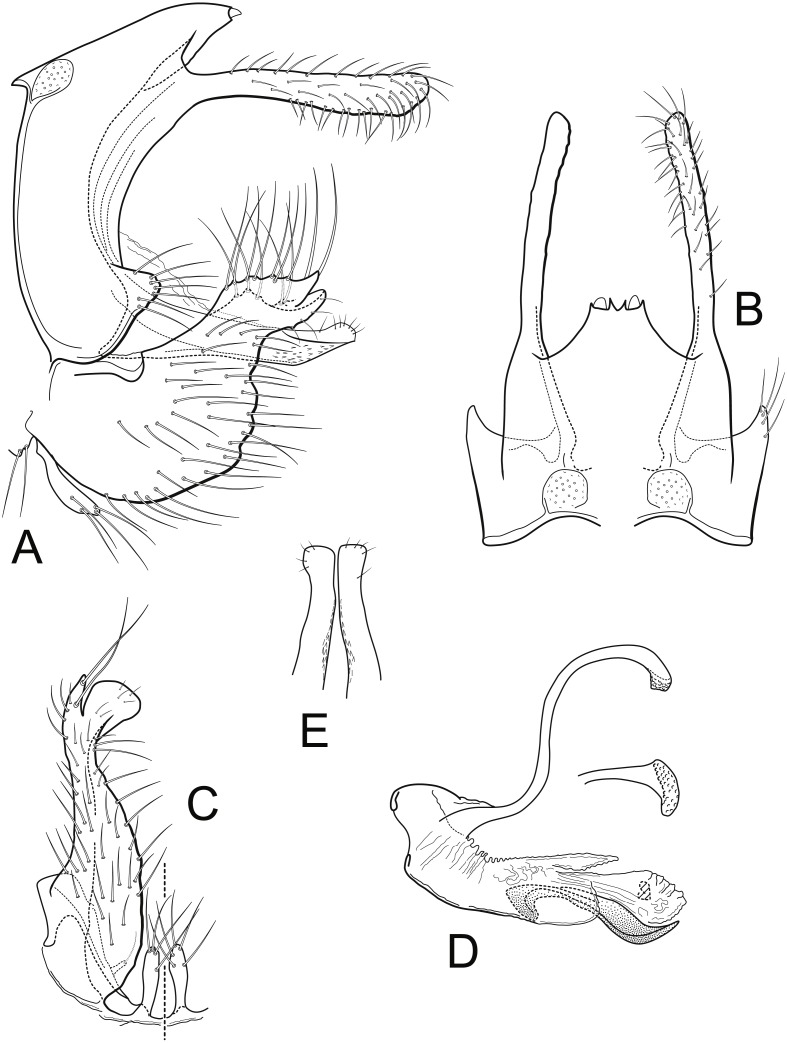
*Nectopsyche spiloma* (Ross). Male genitalia (A) segments IX-X, lateral (B) segments IX-X, dorsal (C) inferior appendage, ventral (D) phallus, lateral, inset: left periphallic process, dorsal (E) apices segment X lateral processes, dorsal. Illustrations by RW Holzenthal.

***Nectopsyche spiloma*(Ross, 1944)**
Figs. 4C, 4D and 5

*Nectopsyche spiloma* was described from Kansas, USA, and is widely distributed in the central and southeastern United States (AL, AR, FL, IL, KS, LA, MO, MS, OK, SD, TX), through Mexico and Central America south to Peru (Holzenthal & Calor, 2017). Previously, the species was reported from Ecuador, but no specific locality information was provided (Flint & Reyes, 1991). Here we provide specific locality records for the species from Ecuador, and provide an updated diagnosis, illustrations of the male genitalia, and color illustration of the adult.

*Nectopsyche spiloma* belongs to a group of species, at least including *N*. *albida* (Walker), *N*. *diarina* (Ross), *N*. *lahontanensis* Haddock, *N*. *stigmatica* (Banks), and *N*. *waccamawensis* Glover and Floyd (Banks, 1914; Glover & Floyd, 2004; Haddock, 1977; Ross, 1944; Walker, 1852). Ross (1944) referred to this group as the *albida*-group, which included at that time, *N*. *albida*, *N*. *diarina*, and *N*. *spiloma*. These species have long narrow preanal appendages; segment IX with a short, subacute ventrolateral protrusion; inferior appendages with a very broad base (“wide flap” of Ross, 1944) and a broadly oval to round apicomesal lobe; broad lateral processes of segment X; and long, narrow, sinuous periphallic processes with capitate, rugose apices. The male genitalia of this species group differ little, and show variation both within and between species, especially in the degree of protrusion and shape of the ventrolateral projection of segment IX, the roundness of the apicoventral lobe and length of the basoventral lobe of the inferior appendage, and the shape of the apex of the lateral process of segment X. The shapes of these various processes and lobes often differ between the right and left sides of an individual specimen and sometimes overlap between species. In combination, the size of the male eyes, the body and forewing color pattern, and the male genitalia can be used to separate the species.

Of the *albida*-group species, the male genitalia and color pattern have been adequately illustrated or described for *N*. *waccamawensis*, *N*. *diarina*, and *N*. *spiloma*. For the latter two species, only the inferior appendage was illustrated, although halftone illustrations of the adult wing coloration of *N*. *spiloma* were provided (Ross, 1944) (fig. 754). A few other species may belong to this group, including *N*. *dorsalis* (Banks), *N*. *gracilis* (Banks), and *N*. *minuta* (Banks) (Banks, 1900; Banks, 1901), but they are inadequately studied to make a reliable assignment at this time. Illustrations of the male genitalia and black and white photographs of the adults of *N*. *gracilis* and *N*. *dorsalis*, respectively, are available (Flint, 1967; Flint, 1991).

Among all of these species, *Nectopsyche spiloma* can be distinguished by a combination of characters, especially in the wing coloration, as first diagnosed in the original description (Ross, 1944). The forewing of *N*. *spiloma* is white with light brown hairs interspersed apically and with a distinct pattern of black hairs in more or less transverse rows. These dark hairs include a diagnostic oval patch of black hairs at the basoventral margin of the forewing (approximately at the base of vein 3A) and one sub-basally in the middle of the forewing (approximately at the base of the thyridial cell). Along the ventral margin of the wing are three approximately linear, diagonal patches of dark hairs that form a chevron pattern when the wings are folded in repose and viewed dorsally. Ross (1944) described the wing pattern as follows: “In this species the black shoulder mark is always present, and the spotting on the anterior three-fourths of the wing is always sparse, with heavy dorsal V-marks.” Among the *albida*-group species, *N*. *lahontonensis* and *N*. *stigmatica* are brown or gray-brown species, with small, scattered patches of lighter setae. *Nectopsyche albida*, *N*. *diarina*, and *N*. *waccamawensis* are white species, with varying degrees of light brown patches or rows of hairs, but without the distinct black spots seen in *N*. *spiloma*. In the male genitalia, the basoventral lobe of the inferior appendage in *N*. *spiloma* is less than half the length of the basal part of the appendage, whereas it is more than half the length in the other species.

### Material examined

**COSTA RICA: San José:** Reserva Biológica Carara, Río Carara in Carara, 9.778°N, 84.531°W, ei. 200 m, 14.iii.1991, Holzenthal, Muñoz, Huisman, ♂, UMSP [illustrated]. **ECUADOR: Esmaraldas:** Río Galera, ca. 1 km S Galera, 00.80747°N, 80.04692°W, el. 20 m, 17.ii.2017, Holzenthal, Ríos-Touma, Huisman, Amigo, 22 ♂, 19 ♀ (UMSP). **Manabi:** Río Camarones, above village of Camarones, 00.11160°S, 80.14442°W, el. 100 m, 19.ii.2017, Ríos-Touma and Amigo, 25 ♂, 11 ♀; Río Tabuga, ca. 4.5 km E Tabuga, 00.08379°S, 80.12458°W, el. 187 m, 18.ii.2017, Holzenthal, Ríos-Touma, Huisman, Amigo, one ♂, two ♀ (UMSP).

## Discussion

Although only six species of *Nectopsyche* have been described in the last 18 years (Holzenthal & Calor, 2017), it is certain that there are still many more to be discovered, especially in the tropical Andes, where studies on the aquatic insect fauna are limited. The existing ecological data are limited to a few studies, but suggest that larvae of this genus are potentially very susceptible to environmental changes. Riparian deforestation can be especially damaging to its populations because of their high dependence on leaf litter (Ríos-Touma, Encalada & Prat Fornells, 2009). Also, habitat loss can have effects on *Nectopsyche* due to its preference for slowly flowing areas and larger substrates (Ríos-Touma, Encalada & Prat Fornells, 2011). Unfortunately, these kinds of impacts in rivers are very common in the Andean valleys (Acosta et al., 2009), where unknown species of *Nectopsyche* likely occur.

## Conclusion

*Nectopsyche spiloma* was reported from Ecuador, but with unspecified locality data (Ríos-Touma et al., 2017). We provide substantiated records of this species from an understudied part of the country. *Nectopsyche paramo* appears to be widespread in the Ecuadorian Andes having been collected from localities that are far away from each other (Pichincha and Bolivar Provinces). Further investigations will be necessary to determine *Nectopsyche* diversity across the high Andes and to assess the differences among populations isolated in different mountain ranges, as observed for other aquatic insects (Finn, Encalada & Hampel, 2016).

## Additional Information and Declarations

### Field Study Permissions

The following information was supplied relating to field study approvals (i.e., approving body and any reference numbers): This study was performed under the Environmental Ministry of Ecuador study permits: 36-2010-IC-FLO/FAU-DPA-MA, 005-15-IC-FAU-FLO-DNB/MA, and MAE-DNB-CM-2016-0045.

### Data Availability

The following information was supplied regarding data availability: Types of the new species and other material examined are deposited in the University of Minnesota Insect Collection, St. Paul, Minnesota, USA (UMSP), the Museo Ecuatoriano de Ciencias Naturales, Insituto Nacional de Biodiversidad, Quito, Ecuador (MECN), and the Museo de Ecología Acuática, Universidad San Francisco de Quito, Ecuador (USFQ).

### New Species Registration

The following information was supplied regarding the registration of a newly described species: Publication LSID: urn:lsid:zoobank.org:pub:D87B7D98-3111-48A1-B100-4835F7179AE2; *Nectopsyche paramo* LSID: urn:lsid:zoobank.org:act:770BCEBD-90BD-478B-B45F-4D4ECA894F8D.
